# Emerging role and therapeutic implications of p53 in intervertebral disc degeneration

**DOI:** 10.1038/s41420-023-01730-5

**Published:** 2023-12-01

**Authors:** Yidian Wang, Shouye Hu, Weisong Zhang, Binfei Zhang, Zhi Yang

**Affiliations:** Department of Joint Surgery, Honghui Hospital, Xi’an Jiaotong University, Xi’an, Shaanxi China

**Keywords:** Cell signalling, Oncogenesis, DNA damage and repair

## Abstract

Lower back pain (LBP) is a common degenerative musculoskeletal disease that imposes a huge economic burden on both individuals and society. With the aggravation of social aging, the incidence of LBP has increased globally. Intervertebral disc degeneration (IDD) is the primary cause of LBP. Currently, IDD treatment strategies include physiotherapy, medication, and surgery; however, none can address the root cause by ending the degeneration of intervertebral discs (IVDs). However, in recent years, targeted therapy based on specific molecules has brought hope for treating IDD. The tumor suppressor gene p53 produces a transcription factor that regulates cell metabolism and survival. Recently, p53 was shown to play an important role in maintaining IVD microenvironment homeostasis by regulating IVD cell senescence, apoptosis, and metabolism by activating downstream target genes. This study reviews research progress regarding the potential role of p53 in IDD and discusses the challenges of targeting p53 in the treatment of IDD. This review will help to elucidate the pathogenesis of IDD and provide insights for the future development of precision treatments.

## Facts

- IDD is considered to be an IVD cell-mediated degeneration process involving molecules, cells and tissues, which impairs the load-bearing capacity of IVD by affecting its tissue composition and biomechanical properties.
- As a transcription factor, p53 is the central hub of the molecular network that controls cell metabolism and survival, and regulates protein expression in various cellular processes.
- In IDD, p53 is activated by multiple stress signals and exhibits different dynamic characteristics, which can lead to different cell fates.
- p53 is involved in various signal transduction pathways in IVD cells, and participates in the IDD process by influencing IVD cell aging, apoptosis, ECM metabolism, and oxidative stress.

## Open questions

- In IDD, what are the changes in the dynamic characteristics of p53 in the face of different stress signals?
- What is the role of p53 in signal transduction and phenotypic changes of IVD cells?
- What are the implications of p53 in the formulation of accurate treatment strategies for IDD?

## Introduction

**Fig. 1:**
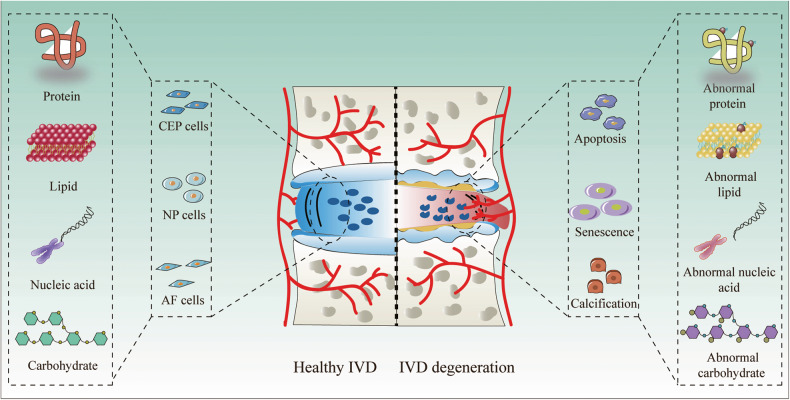
Pathogenic mechanism of IDD. The normal function of an IVD relies on the structural integrity of its organization. During IVD degeneration, aberrant proteins, lipids, carbohydrates, and nucleic acids disrupt cellular homeostasis through apoptosis, senescence, and calcification processes. Consequently, these alterations in IVD composition adversely impact its organizational structure and ultimately compromise its mechanical functionality.

**Fig. 2:**
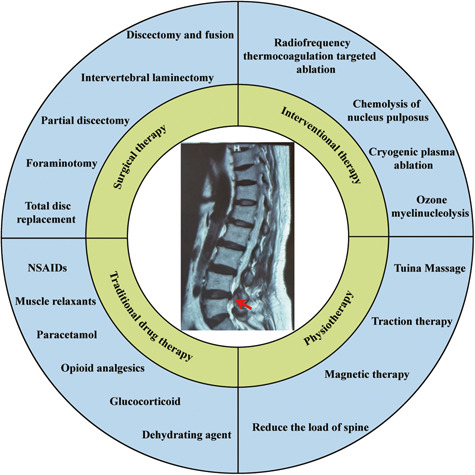
Traditional treatment of IDD. Conventional therapeutic approaches for IDD encompass pharmacotherapy, physical rehabilitation, minimally invasive procedures, and surgical interventions.

Lower back pain (LBP) is a major musculoskeletal disorder that leads to limited mobility and decreased quality of life in elderly individuals worldwide [1]. With the intensification of aging, the overall disability associated with LBP is on the rise globally [2, 3] and is most pronounced in low- and middle-income countries [4]. According to limited data, the one-year prevalence of LBP among adults in Africa and Latin America is 57% and 67%, respectively [5, 6]. The lifetime prevalence rate can be as high as 93% [7]. LBP seriously affects the quality of life of patients and creates a huge economic burden. In the United States, the total cost associated with LBP exceeds $100 billion annually [8], and the cost of spinal surgery in Brazil increased by 540% between 1995 and 2014 [9]. Intervertebral disc degeneration (IDD) is the primary cause of LBP [10]. However, the specific pathogenic mechanisms underlying IDD remain unclear. Currently, IDD is considered a degenerative process involving molecules, cells, and tissues mediated by intervertebral disc (IVD) cells that can lead to significant changes in IVD tissue composition and biomechanical properties, ultimately impairing the ability of IVDs to withstand loads [11] (Fig. 1). Currently, the treatment options for IDD include drugs and surgery, which relieve symptoms and reduce the incidence of disability; however, both treatment options have the disadvantages of multiple complications, high costs, and unknown efficacy [12] (Fig. 2). Neither approach resolves the underlying pathology by terminating the degenerative process of IVDs, and both are only applicable to end-stage disease. Therefore, it is important to further explore the pathogenic factors and related molecular mechanisms of IDD to guide treatment.

**Fig. 3:**
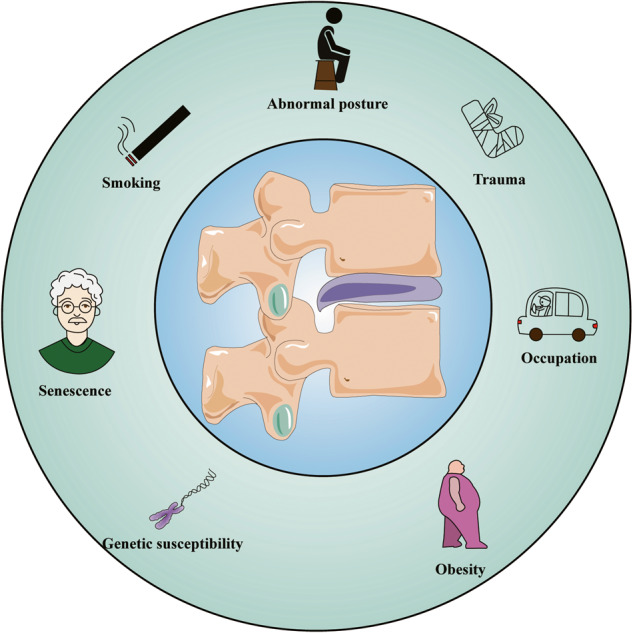
Main pathogenic factors of IDD. Various factors have been associated with the development of IDD, including genetic predisposition, age, obesity, smoking, occupational exposure, trauma, and abnormal nonphysiologic mechanical loads.

**Fig. 4:**
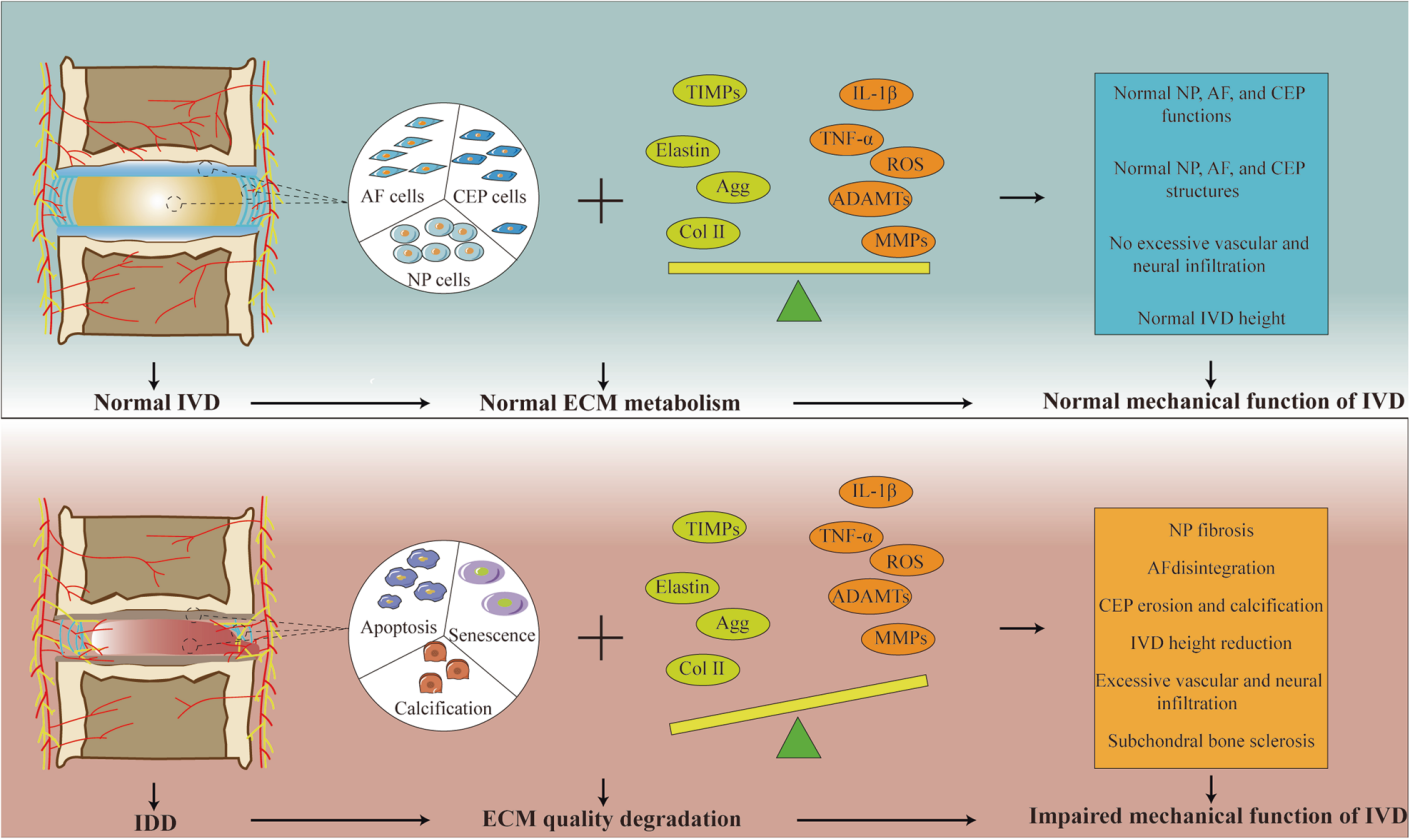
Multiple factors promote IDD progression by influencing ECM metabolism. Top: In a healthy IVD, an adequate number of IVD cells and normal cellular metabolic function are essential for maintaining the integrity of the ECM, which in turn preserves the structural and functional homeostasis of the IVD. Bottom: In degenerating IVDs, a reduction in the IVD cell population due to apoptosis, senescence, and calcification leads to the downregulation of ECM synthesis. Simultaneously, dysfunctional IVD cells exhibit high expression of pro-inflammatory cytokines, ROS, MMPs, and ADAMTS, further accelerating ECM degradation and ultimately deteriorating IVD tissue structure and biological function.

The IVD is a complex avascular connective tissue between the vertebrae, mainly composed of the nucleus pulposus (NP), annulus fibrosus (AF), and cartilage endplate (CEP). Additionally, it connects the spine, cushions spinal pressure, and increases spinal mobility [13–15]. As an age-related, multifactorial disease, the etiology of IDD remains unclear. Genetic susceptibility, age, obesity, smoking, occupational exposure, trauma, and abnormal nonphysiological mechanical loads contribute to its occurrence and progress [16–21] (Fig. 3). The proper mechanical function of the IVD depends on the quality and composition of the extracellular matrix (ECM) [22]. However, in the process of IVD degeneration, a series of internal, external, physical, or chemical factors promote the death and aging of IVD cells, leading to a decrease in the number of functional and viable cells and, thus, a decline in ECM synthesis [23, 24]. At the same time, dysfunctional IVD cells highly express matrix metalloproteinases (MMPs) and A disintegrin and metalloproteinase with thrombospondin motifs (ADAMTS), which further promote ECM degradation [25–27]. The ECM components mainly include collagen (Col) networks and water-bound aggrecan (Agg), which have different proportions in the NP and AF [22]. The ECM in the NP mainly consists of Col II, elastin, and Agg, whereas the AF consists of alternating Col I [11]. The dysfunction of the NP and AF leads to the gradual loss of Col II and Agg, which causes the disappearance of the boundary between the NP and AF, reduces IVD height, and impairs the ability of the IVD to withstand mechanical loads [28]. Structural damage to the AF is conducive to infiltrating blood vessels and nerve endings, further promoting the deterioration of the IVD microenvironment and leading to IVD-derived LBP [29–31]. In addition, as a type of hyaline cartilage mainly composed of proteoglycans and collagen fibers, the CEP plays an important role in the exchange of nutrients and metabolic waste [32]. CEP degeneration can lead to nutritional disorders and metabolic waste accumulation in IVD cells, which helps accelerate IDD [33]. Therefore, various adverse factors lead to IVD cell death and dysfunction, which eventually leads to IDD by affecting ECM metabolism (Fig. 4).

As a transcription factor, p53 is the central hub of the molecular network that controls cell metabolism and survival and is involved in the regulation of protein expression in cell cycle progression, aging, apoptosis, tumor metastasis, DNA repair, reactive oxygen species (ROS) metabolism, and glycolysis [34–36]. Recent studies have suggested that p53 activation and signal transduction are essential for the maintenance of IVD homeostasis. Zhang et al. [37] showed that p53 mRNA expression is significantly upregulated with increasing degrees of IVD degeneration. In addition, p53 knockdown in rats reduced the apoptotic phenotype of IVD cells by downregulating the expression of Bax and caspase 3, suggesting that the inhibition of p53 gene expression may be beneficial for delaying the progression of IDD [37]. In this review, we focus on the activation of p53 signaling in IVD, the signal transduction network, and its multiple biological functions in IVD cells, combined with existing evidence to comprehensively clarify the role of p53 in IDD.

### The origin and structure of p53

In 1979, DeLeo et al. [38] first identified a protein with an apparent molecular weight of 53,000 in chemically induced sarcoma, leukemia, and spontaneously transformed fibroblasts and named it p53. Initially, researchers thought it was an oncogene because its mutant protein was expressed in many cancer tissues [39–41]. However, subsequent studies have demonstrated that wild-type p53 exhibits tumor suppression properties [42, 43]. Many studies have shown that p53 is an important tumor suppressor and plays an important role in the development of neurodegenerative diseases, cardiovascular diseases, diabetes, and osteoarthrosis [44–47].

**Fig. 5:**
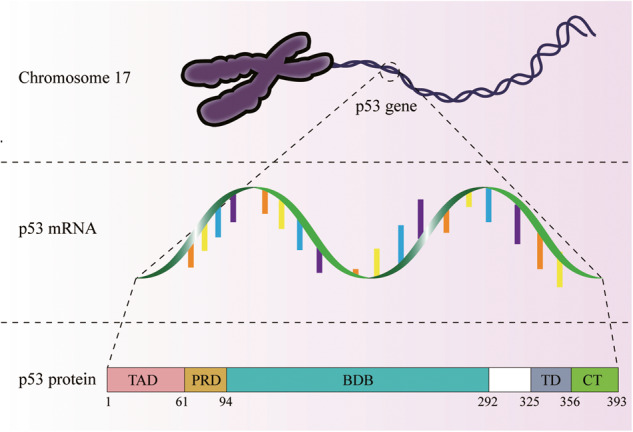
Gene localization and protein structure of p53. Top: Chromosomal localization of the p53 gene. Middle: p53 mRNA. Bottom: p53 protein structure.

The human p53 gene is located on the short arm of chromosome 17, which consists of 11 exons and 10 introns in a 2.2–2.5 Kb gene structure [48, 49]. Transcription of the p53 gene may start from five promoter regions: P0, P1, P1’, P2, and P^in^, with P0, P1, and P1’ located in the first exon and the other two promoters located in introns [50]. From the P0, P1, and P1’ promoters, 2.5 Kb mRNA can be synthesized, encoding a full-length p53 protein containing 393 amino acids [50, 51]. The p53 protein consists of five main structural regions: the N-terminal transactivation domain, the proline-rich domain, the DNA-binding domain (DBD), the tetramerization domain, and the C-terminal domain [52] (Fig. 5). Detailed descriptions of the functions of major p53 structural regions have been documented previously [53–57].

### p53 and IDD

As a critical intracellular transcription factor, p53 is activated by cellular stress and promotes cell cycle arrest, DNA damage repair, and cell death through transcription-dependent and -independent pathways [58]. In these metabolic processes that determine the fate of cells, p53 often acts as a “double-edged sword,” complicating its role in specific diseases [59–61]. According to microarray and bioinformatics studies, p53 is a hub gene in a level-III-specific network, indicating that p53 may be involved in the progression of IDD [62, 63]. Analysis of degenerated IVD specimens from patients with lumbar disc herniation and rats showed that vascular endothelial growth factor and p53 expression increased in degenerated IVD tissues. The expression rate was significantly higher in tissues with vascular infiltration than those without, suggesting that they may be involved in late-stage vascularization and infiltration, accelerating IDD [64, 65]. Significantly increased p53 expression is involved in IVD cell apoptosis, aging, and ECM degradation by activating downstream target genes [37, 66–71]. These studies indicate that elevated p53 levels are important in promoting IDD progression. Xiong et al. [72] demonstrated that high p53 expression can protect nucleus pulposus cells (NPCs) from low cell viability and ECM degradation induced by low glucose concentrations, indicating that p53 can be used as a protective agent for NPCs under certain conditions. These studies suggest that p53 is essential for maintaining IVD homeostasis, and changes in its expression are directly involved in the progression of IDD.

### p53 dynamics

#### General characteristics of p53 dynamics

**Fig. 6:**
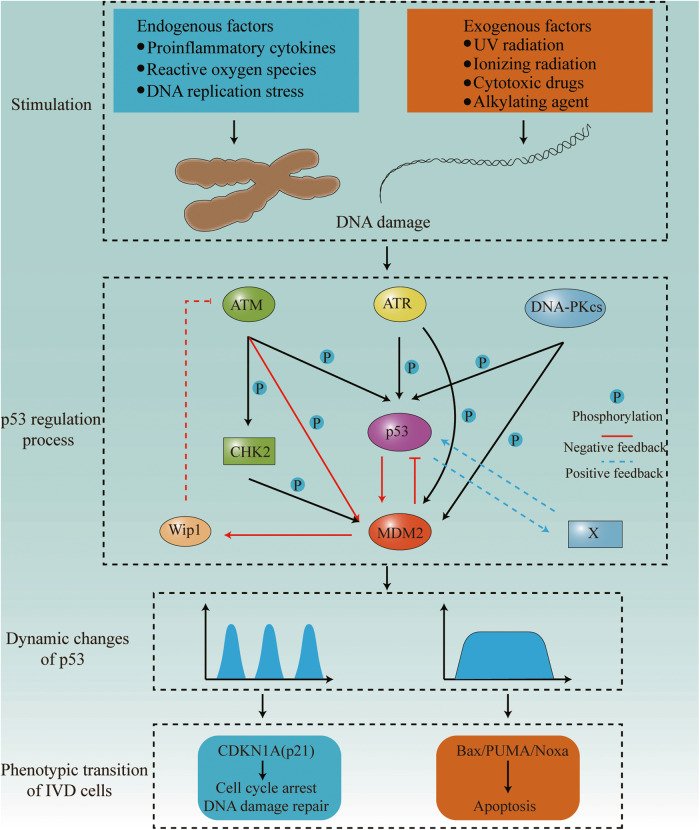
Feedback regulation of p53 dynamics and cellular processes corresponding to different dynamics. In the process of IVD degeneration, various intracellular and extracellular stress signals activate p53 by inducing DDR. Activation produces different dynamic changes in p53 by regulating p53-MDM2 and p53-Wip1-ATM negative feedback loops and potential positive feedback loops. These distinct alterations in p53 kinetics elicit diverse phenotypic modifications in IVD cells, thereby ultimately contributing to the progression of IDD.

Traditionally, cell signaling dynamics refers to the temporal expression pattern of active signaling molecules, which is an important mechanism by which cells respond to complex stimuli [73, 74]. Dynamic regulation enables signaling molecules to respond to several stimuli and coordinate the activation of different and even conflicting downstream pathways to increase the specificity and function of signaling pathways [75]. As one of the most important tumor suppressors, p53 maintains genomic integrity by regulating the expression of many downstream target genes essential for relieving stress and regulating cell fate [76, 77]. The dynamics of p53 depend on the type, intensity, and duration of stimulation and show different patterns, including pulse, sustained platform, monotonic increase, and biphasic dynamics [73, 78]. The most intuitive dynamic change in p53 is a change in p53 protein abundance, which may be related to the selective activation of downstream target genes [79, 80]. Due to differential p53 thresholds for the activation of genes involved in cell cycle arrest and apoptosis, low levels of p53 primarily induce gene expression such as p21, leading to cell cycle arrest, while relatively higher levels of p53 tend to activate genes like Bax, triggering apoptosis [81]. In non-stressed cells, p53 remains low because of its rapid degradation. However, p53 can perform spontaneous transient pulses in non-stressed cells to cope with spontaneous DNA damage [82, 83]. Stressed cells can convert the p53 dynamic models induced by different stimuli into specific types of downstream responses [84] (Fig. 6). Ultraviolet (UV) light induces prolonged p53 pulses that increase in width and height with increasing UV doses, ultimately leading to apoptosis [85]. In contrast, ionizing radiation (IR) induces differential p53 kinetics. Low doses of IR induce low-frequency transient p53 pulses, which can lead to cell cycle arrest and DNA damage repair [86, 87]. However, high doses of IR can induce high-frequency p53 pulses and apoptosis [88]. In addition to stress types, there are significant differences in p53 kinetics among different cell types [89], reflecting that only cell-level p53 dynamics can accurately reflect the regulation of p53-mediated cellular signal transduction. Furthermore, p53 kinetics behave differently in different tissues. The small and large intestines are relatively insensitive to p53 levels. In contrast, the lymphoid organs are more sensitive to radiation, which consistently induces p53 expression [90]. The variability of p53 dynamics originates from the fact that p53 can activate the expression of genes that promote cell injury repair and death and induce the expression of genes that regulate their own gene expression and protein stability, leading to the formation of feedback loops and resulting in dynamic changes in p53 levels [91, 92]. Recent studies have shown that p53-MDM2 and p53-Wip1-ataxia telangiectasia-mutated (ATM) negative feedback loops are key factors in the synergistic control of p53 excitability, with the former being the topological basis of excitability in p53 kinetics and the latter regulating the sensitivity of p53 excitable responses [92–96]. However, it is difficult to explain the continuous p53 pulse that induces apoptosis through these two negative feedback loops. Therefore, a few scholars believe that a p53 positive feedback loop can induce a sustained p53 pulse to maintain high p53 levels [97]. For example, retinoid-related orphan receptor-α can stabilize p53 and activate p53 transcription in a HAUSP/Usp7-dependent manner, which has been shown to play a key role in persistent p53 pulses induced by DNA damage from extensive stimulation [97, 98]. Thus, positive and negative feedback regulation of p53 allows for the coordination of transient and continuous p53 pulses, constituting a flexible and effective regulatory network. In addition to regulating protein levels, p53 kinetics are characterized by changes in p53 protein activity, subcellular localization, post-translational modifications, and interactions among all p53 kinetic characteristics [96, 99, 100]. Therefore, p53 dynamics do not exhibit a simple linear relationship but a multifaceted dynamic network.

#### Dynamics of p53 in IDD

p53 is involved in maintaining IVD microenvironment homeostasis [68, 101]. Compared with that in normal IVDs, the expression of p53 in degenerative IVDs is significantly increased, and the degree of increase is related to the grade of degeneration [37, 64–66, 71, 102]. In addition, multiple stimuli can lead to the upregulation of p53 levels, including oxidative stress, inflammatory responses, high glucose levels, and abnormal mechanical loading [101, 103–105]. However, the exact changes in p53 temporal dynamics under these stress conditions remain unclear. As a signaling molecule, p53 is continuously transported between the nucleus, cytoplasm, and mitochondria; this change in subcellular localization reflects the spatial dynamics of p53 [106, 107]. p53 is mainly located in the nucleus, which binds to the promoter or enhancer of target genes to regulate gene expression [58]. However, a small portion of the p53 protein is located in the mitochondria to perform its non-transcriptional functions [108]. Analysis of mitochondrial gene expression patterns showed that p53 was significantly upregulated in the mitochondria of AF cells with a higher grade of degeneration [102]. In addition, the expression of p53 in the mitochondria of NPCs increased under a compression load, which led to a significant upregulation of apoptosis in NPCs [109]. Therefore, the mitochondrial translocation of p53 may promote apoptotic activity. In addition, as one of the kinetic characteristics of p53, acetylation is an important modification required for p53-mediated cell senescence and apoptosis [110, 111]. Zhang et al. [112] showed that the acetylation level of p53 in NPCs was significantly increased under high glucose levels, accompanied by excessive senescence and NPC apoptosis. Although many studies have explored the role of p53 in IDD, our understanding of p53 dynamics is limited to changes in p53 protein levels. However, the biological significance of the differences in temporal dynamics, subcellular localization, and post-translational modification of p53 in response to different stimuli remains unclear. Different types of p53 kinetic changes and their interactions are essential for the regulation of specific cellular injury responses. Therefore, further exploration of the characteristics of p53 kinetics will help us understand its potential role in IDD.

#### Activation of p53

p53 is activated under various stress conditions, including DNA damage, inflammatory responses, hypoxia, high-glucose conditions, and oxidative stress [34, 113, 114]. p53 is extremely sensitive to DNA damage; therefore, it is called the “genome guardian” [113]. DNA damage response (DDR) consists of three key stages: damage perception, signal cascade, and damage repair [115]. Damage perception is accomplished by several kinases, including ATM, ataxia telangiectasia Rad3-related protein (ATR), and DNA-dependent protein kinase catalytic subunits (DNA-PKcs) [116, 117]. These kinases detect damage and signal to p53 through the Mre11-Rad50-NBS1 protein complex [96, 117]. ATM mainly responds to double-strand breaks, ATR mainly responds to single-strand breaks, and DNA-PKcs respond to double- and single-strand breaks [96, 118, 119]. In the resting state, ATM and ATR are inactive dimers recruited and activated by intermolecular autophosphorylation and homodimer dissociation following DNA damage [115, 116]. ATM, ATR, and DNA-PKcs can stabilize p53 by phosphorylating p53 and MDM2, and ATM-dependent MDM2 phosphorylation can accelerate MDM2 degradation, indirectly promoting p53 activation [96]. The occurrence and development of IDD are closely associated with DNA damage [120, 121]. In DNA repair-deficient Ercc1^-/Δ^ mice, excessive DNA damage promoted IVD cell senescence and ECM degradation, accelerating IDD [122, 123]. Potential genotoxic stressors such as ionizing radiation and smoking can accelerate IDD in mice by inducing DNA damage [124, 125]. In addition, excessive ROS in degenerative IVDs is another source of DNA damage [126–128]. Recently, Han et al. [129] directly exposed human NPCs to the DNA-damaging agent cisplatin. In vitro, cisplatin-induced rapid phosphorylation of ATM in human NPCs led to ECM degradation and cell senescence; however, these effects were weakened after treatment with ATM-specific inhibitors. ATM knockdown reduced IVD cell senescence and ECM degradation in an accelerated senescent Ercc1^-/Δ^ mouse model in vivo. This suggests that DNA damage affects the progression of IDD by activating ATM to promote the activation of p53. In addition, non-physiologically intense cyclic mechanical loading increases DNA damage in NPCs, activating the p53/p21/retinoblastoma protein (Rb) pathway and mediating premature NPC senescence [130]. Oxidative stress activates p53 through redox signaling and DDR induction, and oxidative post-translational modifications of p53 cysteine residues are thought to be associated with this process [131, 132]. In addition, redox signaling activates p53 through Jun N-terminal kinase (JNK) [133]. In IDD, treatment of human NPCs with H_2_O_2_ significantly increases intracellular ROS and p53 expression, in turn inducing cellular senescence by activating p21 [101]. In addition, a few inflammatory cytokines directly or indirectly promote p53 activation. IL-1β can upregulate the expression of p53 and its downstream genes, leading to cell cycle arrest and PUMA/Bax-mediated apoptosis of neural progenitor cells [134]. In ischemia/reperfusion-associated acute neuronal injury, IL-1β induces p53-dependent apoptosis by promoting NF-κB nuclear translocation [135]. Similarly, IL-1β can induce the degradation of NPC ECM through the NF-κB/p53 pathway, promoting the progress of IDD [136]. These studies suggest that multiple factors can directly or indirectly activate p53, initiating p53-related downstream responses that affect IDD progression.

#### Signal transduction of p53

**Fig. 7:**
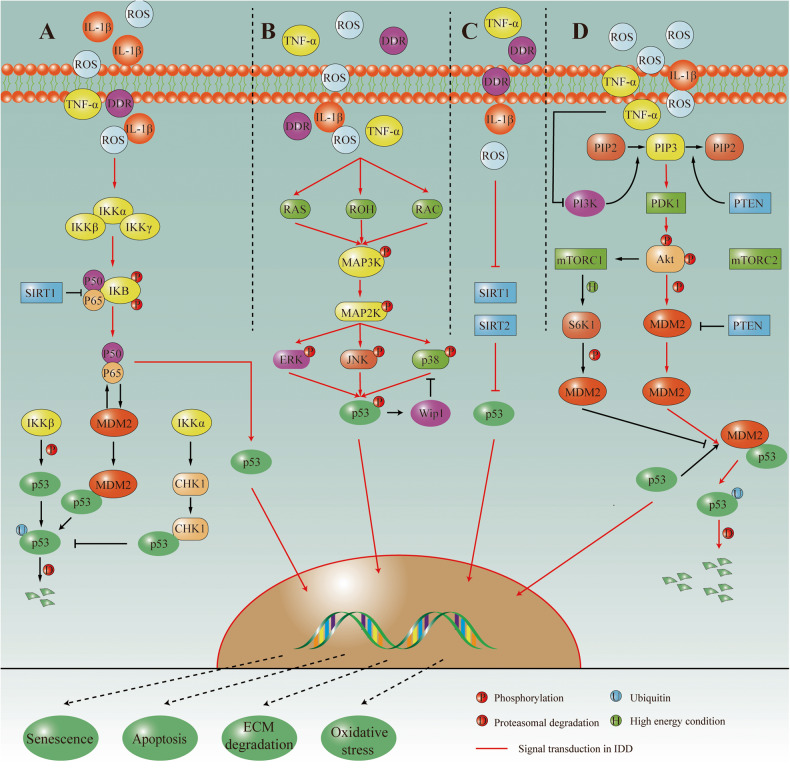
Crosstalk between different signal pathways and p53. **A** Crosstalk between the NF-κB signaling pathway and p53. **B** Crosstalk between the MAPK/ERK signaling pathway and p53. **C** Crosstalk between the SIRT signaling pathway and p53. **D** Crosstalk between the PI3K-Akt signaling pathway and p53.

As a transcription factor that responds to stress signals, p53 participates in complex signal transduction in cells [44]. Due to the central role of p53 in cellular stress and its involvement in various cellular processes such as DNA damage repair, cell cycle arrest, senescence, apoptosis, and metabolic regulation [137, 138], it can be speculated that the functional connections between p53 and other signal transduction molecules are diverse and regulate phenotypic changes in IVD cells by acting on dynamic signaling networks with complex feedback loops comprising different signaling pathways (Fig. 7). However, the exact molecular mechanisms of p53-mediated signaling remain elusive, and further studies are needed to elucidate the additional signaling pathways regulated by p53 and determine their mechanisms.

### PI3K-Akt

#### Activation of the PI3K-Akt signaling pathway

The phosphatidylinositol 3-kinase (PI3K)/protein kinase B (PKB/Akt) signaling pathway is a central pathway that regulates cell survival, proliferation, motility, and metabolism [139]. PI3Ks are a group of plasma membrane-associated lipid kinases with a common substrate, Akt [140]. Akt comprises a group of serine/threonine protein kinases with three isoforms, Akt1, Akt2, and Akt3 that are activated in response to upstream PI3Ks and regulate cellular metabolic processes by activating downstream target genes [141]. Various stimuli, including growth factors, cytokines, hormones, and stress, initiate PI3K/Akt signal transduction [142]. These molecules promote the activation of PI3K by activating the corresponding receptor tyrosine kinases, and activated PI3K converts phosphatidylinositol-4,5-bisphosphate (PIP2) to phosphatidylinositol-3,4,5-trisphosphate (PIP3) [143]. After generation, PIP3 acts as a second messenger to simultaneously recruit 3-phosphoinositide-dependent protein kinase 1 (PDK1) and Akt to the plasma membrane, such that PDK1 phosphorylates the threonine 308 site of the Akt protein, leading to its partial activation [144, 145]. Subsequently, mTOR complex 2 (mTORC2) phosphorylates the serine 473 site of Akt, resulting in its complete activation [146]. During this process, phosphatase and tensin homolog (PTEN) negatively regulate the PI3K/Akt signaling pathway by converting PIP3 to PIP2 [147].

#### Crosstalk between the PI3K-Akt signaling pathway and p53 in IDD

The PI3K/Akt signaling pathway functions through a variety of substrates, including forkhead box O (FOXO), glycogen synthase kinase 3 (GSK3) and mTORC1 [147, 148]. This pathway interacts with p53 through multiple mechanisms [149, 150]. When this pathway is activated, Akt leads to MDM2 translocation to the nucleus by phosphorylating serine 166 and 183 sites on MDM2. This promotes the ubiquitin-dependent proteasomal degradation of p53, thereby preventing ischemic injury-induced neuronal apoptosis and oxidative stress-induced cellular senescence in mice [151]. In addition, the p53-induced caspase cascade accelerates the degradation of MDM2 and Akt, thereby forming a negative feedback loop [152]. Regulation of p53 by the PI3K/Akt pathway is mediated by its downstream target, mTORC1. Under high-energy conditions, PI3K/Akt activates mTORC1, which in turn promotes MDM2 phosphorylation via the Akt/mTORC1/S6K1 signaling axis, leading to the inhibition of MDM2-mediated, ubiquitin-dependent p53 degradation and increasing the stability of p53 [153]. In addition, PTEN promotes the stability of p53 through the feedback inhibition of Akt activation and the downregulation of MDM2 transcription [154, 155]. In IDD, the PI3K/Akt pathway was significantly inhibited after NPCs were treated with IL-1β, which led to increased expression of p53 and induced cell senescence through the p53/p21 signal axis. In contrast, the expression of p53 was downregulated after pretreatment with a PI3K/Akt pathway activator [156]. Gong et al. [157] showed that bone morphogenetic protein-7 (BMP-7) attenuates replicative senescence in human NPCs by activating the PI3K/Akt pathway to downregulate the p53/p21 signaling axis, whereas treatment with the PI3K/Akt pathway inhibitor LY294002 partially inhibits the protective effect of BMP-7 on cellular senescence. These studies suggest that the activation of the PI3K/Akt signaling pathway inhibits p53 activation, which protects against IVD cell degeneration. Complex feedback regulation occurs between the PI3K/Akt signaling pathway and p53. Therefore, further exploration of the regulatory mechanism between the PI3K/Akt signaling pathway and p53 could help researchers elucidate the pathogenesis of IDD and develop new therapeutic approaches.

### NF-κB

#### Activation of the NF-κB signaling pathway

NF-κBs are a group of transcription factors that bind to enhancer elements of the immunoglobulin κ light chain of activated B cells, which has two different activation mechanisms, classical and non-classical [158]. The activation of classical pathways plays an important role in tumors, neurodegenerative diseases, cardiovascular diseases, and musculoskeletal diseases by regulating various phenotypic changes, such as inflammation, immune response, cell proliferation, survival, and differentiation [11, 159–161]. Molecular cloning revealed that the human NF-κB transcription factor consists of five DBDs: NFKB1 (p50), NFKB2 (p52), REL (c-REL), RELA (p65), and RELB, the latter three of which possess transactivation domains [162]. In the classical signaling pathway, NF-κB is formed by heterologous DNA-binding dimers such as p65 and p50, which bind to their specific inhibitor IκB and are present as inactive complexes in the cytoplasm of most quiescent cells [163, 164]. IκB mainly consists of IKKα (IKK1), IKKβ (IKK2), and the regulatory subunit IKKγ, which forms the IKK complex with IKKα/IKKβ as a dimer [143, 165]. When cells are externally stimulated, IκB is phosphorylated and subjected to ubiquitin-dependent proteasomal degradation. This causes NF-κB to induce κB factor translocation to the nucleus and activate downstream target genes to regulate cell function [143, 164]. Meanwhile, NF-κB induces the expression of negative regulators such as IκBα, A20, and p105 to form a negative feedback regulatory mechanism [158, 166].

#### Crosstalk between the NF-κB signaling pathway and p53 in IDD

In the cellular signal transduction system, many intersections occur between the p53 and NF-κB pathways, indicating a wide range of mutual regulation between them. Regarding the ability to stimulate transcription, both p53 and NF-κB use the same histone acetyltransferase (p300/CBP) to promote the rapid transcription of target genes by acetylating related transcription factors and histones [167]. When both p53 and NF-κB respond to stress signals, the p65 subunit and p53 negatively regulate each other’s activities by competing for a limited protein pool of p300 and CBP coactivators [168–170]. The cross-transcriptional interference between p53 and NF-κB is caused by the direct interaction between these two transcription factors through the dimerization or tetramerization domain, leading to the inhibition of transcriptional activity independent of p300/CBP [171]. Another regulatory node in the p53 and NF-κB pathways is MDM2. MDM2 is a common downstream target gene of p53 and NF-κB signaling pathways, leading to MDM2 upregulation and p53 downregulation [172]. In addition, MDM2 acts as a cofactor for NF-κB, binding to the promoter binding site of its target gene and playing a role in activating NF-κB in sterile tissue inflammation [173]. As another regulatory node, an alternative reading frame (ARF) can bind and inhibit MDM2, thereby increasing p53 levels and activity [174, 175]. At the same time, ARF induces the formation of an ATR/checkpoint kinase 1 (CHK1) complex, which phosphorylates and inhibits NF-κB transcriptional capacity [176, 177]. In addition, the IKK protein family is associated with NF-κB activation and regulates p53 activity. Xia et al. [178] showed that IKKβ can phosphorylate the serine 362 and 366 sites of p53, leading to p53 recruitment by β-TrCP1 and ubiquitin-dependent degradation, thus reducing the level and activity of p53. Recent studies have reported that IKKα, another member of the IKK protein family, can induce the activation of CHK1 and promote the formation of a CHK1/p53 complex, which leads to the phosphorylation and activation of p53 [179]. In addition, IKKα triggers the formation of p300/p53 and CBP/p53 complexes, mediating UV-associated apoptosis by inducing p53 acetylation [180]. The above studies suggest that NF-κB activation usually inhibits p53 function and vice-versa; therefore, their relationship is antagonistic. p53 and NF-κB can synergistically regulate cellular processes under certain circumstances. In colon cancer cells containing wild-type p53, p53 activation induces nuclear translocation of the p65 subunit of NF-κB, leading to apoptosis, whereas a loss of NF-κB activity abolishes p53-induced apoptosis [181]. This suggests that NF-κB is essential for p53-mediated cell death in specific states. In addition, recent studies suggest that NF-κB and p53 may play a synergistic agonistic role in the regulation of pro-inflammatory responses. The coactivation of NF-κB and p53 leads to a significant elevation in the expression of pro-inflammatory genes in human macrophages, including pro-inflammatory cytokines and chemokines [182]. Zhang et al. [71] showed that the expression of p-p65 and p53 was significantly higher in degenerative NPCs than in normal human NPCs. In vitro, IL-1β significantly increased the expression of MMPs and ADAMTS by activating the NF-κB/p53 signal axis and decreased the expression of Agg and Col II, resulting in a decrease in ECM content [71]. In addition, both pro-inflammatory cytokines (IL-1β and TNF-α) and ROS can activate p53 by promoting NF-κB expression and nuclear transcription, inducing IVD cell senescence through the p53/p21 pathway, while the inhibition of NF-κB activity attenuates p53-induced cell senescence [68, 105, 183, 184]. This suggests that the coactivation of NF-κB and p53 may promote IDD progression. These studies suggest a complex network of mutual regulation between NF-κB and p53. Different stimuli and cell types may determine the outcome of regulation between NF-κB and p53. However, the crosstalk between NF-κB and p53 in IDD requires more studies to characterize further.

### MAPK/ERK

#### Activation of the MAPK/ERK signaling pathway

The mitogen-activated protein kinase (MAPK)/extracellular signal-regulated kinase (ERK) signaling pathway plays an important role in the development of tumors, neurodegenerative diseases, infectious diseases, and musculoskeletal disorders by regulating cell survival, death, differentiation, proliferation, and metabolism [143, 185–187]. The complete MAPK cascade is divided into three modules: MAPK kinase (MAPKKK), MAPK kinase (MAPKK), and MAPK, which sense external stimuli and are activated by sequential phosphorylation [188]. Activated MAPKs phosphorylate many specific downstream substrates, leading to transcriptome and proteome reprogramming [189]. The existence of this sophisticated three-layer “core signal module” structure can associate different extracellular stimuli with a wide range of intracellular responses [190]. In mammals, multiple MAPK cascades have been identified, including ERK1/2/3/4/5/7/8, JNK1/2/3, p38 α/β/γ/δ, and nemo-like kinase [187, 188, 191, 192]. Among these, ERK is mainly activated by growth factors and insulin, whereas JNK and p38MAPK are mainly activated by environmental stress [193–196].

#### Crosstalk between the MAPK/ERK signaling pathway and p53 in IDD

Both MAPKs and p53 are activated by cellular stress, and their crosstalk controls the balance between cell survival and metabolism [197]. MAPKs control the activation of p53 by phosphorylation in response to the DDR [198, 199]. In ovarian cancer cells, cisplatin treatment mediates p53 serine 15 phosphorylation by activating ERK1/2 in response to DDR-induced apoptosis, whereas the selective MEK1 inhibitor PD98059 reduces p53 serine 15 phosphorylation and apoptosis [200]. Similarly, in human non-small-cell lung cancer (NSCLC) cells, the activation of JNK phosphorylates p53 at serine 15 and increases its stability by reducing the interaction between p53 and MDM2, thereby inducing G2/M arrest and cancer cell apoptosis [201]. In addition, activated JNK is directly involved in the phosphorylation of p53 at serine 20 and threonine 81, leading to an increase in p53-dependent transcriptional activity in response to DDR and stress inducers; this is inhibited in JNK knockout cells [202–204]. Many studies have shown that p38 can play an important role in activating p53 through phosphorylation. p38 can promote p53 activation and stabilization through the downregulation of MDM2 and phosphorylation of p53-serine 15 in response to apoptosis induced by hypoxia, carcinogenic toxicants, and UV exposure, which can be attenuated by the p38 inhibitors SB203580 and SB202190 [205–207]. In normal human keratinocytes, activated p38 phosphorylates p53 at serine 15, reducing p53 nuclear transcription and reducing UV-induced apoptosis [208]. This suggests that different stimuli and cell types affect the phosphorylation of p53 serine 15. In addition to serine 15, activated p38 phosphorylates p53 serine at sites 20, 33, 46, and 389, activating p53 [202, 209–211]. MAPKs can regulate p53 activity, and p53 can regulate MAPKs by activating downstream target genes. Wip1, a serine/threonine phosphatase, is induced by UV radiation in a p53-dependent manner in response to DNA damage [212]. In NSCLC, Wip1 promotes the expression of stemness-related proteins and cancer stem cell properties by inhibiting p38 activity [213]. Other studies have shown that Wip1 selectively reduces p38 activity by dephosphorylating conserved threonine residues [214]. In addition, other transcription products of p53, members of the bispecific protein phosphatase family, including mitogen-activated protein kinase phosphatase 1 (Mkp1), dual specific phosphatase 2 (DUSP2), and DUSP5, have been shown to inhibit the activities of ERK, JNK, and p38 [215, 216]. In IDD, both IL-1β and TNF-α can increase the phosphorylation of ERK, JNK, and p38 in NPCs, activating p53 and inducing cellular senescence through the p53/p21 pathway [68, 105]. Similarly, advanced oxidative protein products (AOPPs) can increase ERK, JNK, and p38 phosphorylation levels in AF cells and induce cellular senescence through the p53/p21 pathway, whereas intervention with pathway inhibitors ameliorates AOPP-induced senescence in AF cells [70]. Ge et al. [217] showed that syndecan-4 promotes IDD progression by affecting NPC function. Mechanistically, syndecan-4 overexpression significantly increases the expression of JNK, leading to p53 activation and promoting IDD progression by downregulating Col II and Agg expression and upregulating Col X expression. These phenomena were significantly improved when the cells were treated with the JNK inhibitor SP600125. This evidence suggests that complex regulatory mechanisms exist between MAPKs and p53 and that activating MAPK signaling pathways may accelerate IDD progression by promoting p53 activation and stabilization.

### SIRT

#### Activation of the SIRT signaling pathway

The sirtuin (SIRT) protein family is an important gene-silencing complex composed of seven subtypes of type III histone deacetylase [218]. These protein families have highly conserved nicotinamide adenine dinucleotide^+^ (NAD^+^)-binding domains and catalytic functional domains, and each SIRT has different sequences and lengths of the N-terminal and C-terminal domains [219]. SIRT family proteins are functionally diverse, with SIRT1–SIRT3 having strong deacetylase activity and SIRT4–SIRT7 having weaker activity, which allows them to act specifically on different substrates depending on the biological processes they are involved in [143]. In addition, the SIRT protein family has different subcellular localizations: SIRT1, SIRT6, and SIRT7 are mainly located in the nucleus; SIRT3, SIRT4, and SIRT5 are distributed in the mitochondria; and SIRT2 is mainly present in the cytoplasm [219, 220]. SIRT regulates multiple cellular processes, including DNA repair, glucose metabolism, cell survival, inflammation, and senescence, through upstream and downstream signaling pathways and plays an important role in various diseases [221–223].

#### Crosstalk between the SIRT signaling pathway and p53 in IDD

The SIRT family of proteins regulates transcription factors, leading to complex regulatory interactions with p53 [224]. SIRT1, SIRT6, and SIRT7 are primarily located in the nucleus and interact with histones and transcription factors [225]. SIRT1 is the most widely studied member of the SIRT family [226–228]. SIRT1 negatively regulates the biological function of p53 by deacetylating the p53 gene, reducing p53-induced cell growth inhibition, cell cycle arrest, senescence, and apoptosis [229–233]. However, SIRT1 cannot inhibit all p53-mediated biological activities [234]. In contrast, SIRT1 acts synergistically with p53 to amplify the pharmacological effects of polydatin [235]. In addition, SIRT6 and SIRT7 can reduce p53-induced cellular phenotypic changes by deacetylating p53 [236, 237]. Unlike SIRT1, SIRT6, and SIRT7, SIRT2 is mainly located in the cytoplasm and deacetylates several cytoplasmic substrates, including tubulin [238]. SIRT2 is present in the nucleus and is associated with the deacetylation of histones and several transcription factors [239, 240]. SIRT2 induces p53 deacetylation at lysine 382, decreasing p53 transcriptional activity [241–243]. These studies indicate that SIRT2 activation regulates the activity of p53 and vice-versa [244]. SIRT2 expression significantly increases in senescent cells and decreases after p53 knockdown, suggesting that p53 may affect SIRT2 expression. Chromatin immunoprecipitation has revealed a p53 binding site in the SIRT2 promoter, indicating that it is regulated by p53 [244]. SIRT3, SIRT4, and SIRT5 are mainly localized in the mitochondria. In Alzheimer’s disease, SIRT3 attenuates p53-mediated neuronal apoptosis and mitochondrial dysfunction by inhibiting the acetylation of p53 lysine 320 [245]. In addition, SIRT5 interacts with p53 to inhibit its transcriptional activity. Mass spectrometric analysis showed that SIRT5 mediates the desuccinylation of p53 at lysine 120, resulting in the inhibition of p53 activity and p53-mediated apoptosis [246]. SIRT4 may play different regulatory roles in p53 activity. SIRT4 promotes p53 phosphorylation by inhibiting glutamine metabolism, which inhibits pancreatic ductal adenocarcinoma progression [247]. The modulation of p53 by SIRT is observed in IDD [248, 249]. In a high-glucose environment, SIRT1 activation inhibits p53 acetylation, which protects NPCs from high glucose-induced apoptosis and senescence [112]. In addition, CEP degeneration plays an important role in IDD associated with nutritional disorders [250]. Under sublethal oxidative stress, SIRT1 overexpression significantly reduced p53 acetylation and alleviated oxidative stress-induced senescence in human CEP cells by inhibiting the p53/p21 pathway [251]. In addition, SIRT2 mitigated oxidative stress and cellular senescence by inhibiting the IL-1β-induced p53/p21 pathway, thereby preventing IDD progression [252]. These studies suggest that SIRT1 and SIRT2 can reduce apoptosis and senescence in IVD cells and delay the progression of IDD by inhibiting the activity of p53. However, our understanding of the regulatory effects of other members of the SIRT protein family on p53 is limited, and further studies are needed.

## The role of p53 in IDD

### p53 and apoptosis

Apoptosis is the process by which cells stop growing and dividing, ultimately leading to cell death. Apoptosis is divided into endogenous and exogenous apoptosis; the former is known as mitochondrial apoptosis [253]. As key molecules in inducing endogenous apoptosis, Bcl-2 family proteins play a key role in mitochondrial apoptosis by regulating the permeability of the outer mitochondrial membrane and the release of cytochrome c [254]. Based on the modular structure of their homology domain, Bcl-2 family proteins can be divided into pro-apoptotic (Bak, Bax, and Bok) and anti-apoptotic proteins (Bcl-XL, Bcl-2, Bcl-w, and Mcl-1), the interaction between which is essential for maintaining the balance between cell death and survival [255, 256]. p53 induces transcription-dependent apoptosis through the transcriptional activation of various proapoptotic target genes, including Bak, Bax, and PUMA [257]. In addition to transcription-dependent apoptosis, p53 mediates transcription-independent apoptosis [258]. Nuclear magnetic resonance spectroscopy (NMR) shows that the H2 α-helix, L1, and L3 loops in the DBD of p53 bind directly to Bak, enhancing its oligomerization and leading to outer mitochondrial membrane permeability and cytochrome c release. In contrast, point mutations in these regions significantly weaken the ability of p53 oligomeric Bak to induce transcription-independent apoptosis [259, 260]. In addition, Bcl-XL inhibits Bak/Bax homo-oligomerization and apoptosis by forming an inhibitory complex with Bak and Bax [258]. The DBD of p53 can directly bind to Bcl-xL and release Bak and Bax, thereby enhancing the homo-oligomerization of Bak/Bax; this is applicable to Bcl-w, Mcl-1, and Bcl-2 [261–265]. In addition, PUMA is a direct target of p53-mediated mitochondrial apoptosis [266]. Using isothermal titration calorimetry, Han et al. [267] showed that PUMA binds to the DBD of p53 and thus induces apoptosis by a transcriptionally non-dependent mechanism. In IDD, basal p53 expression [37] and phosphorylated and acetylated p53 levels are significantly elevated in degenerative IVD cells [112, 268], leading to PUMA and Bax accumulation and mitochondrial dysfunction, which in turn leads to apoptosis [104, 269]. In addition, compression loading increases the expression of p53 in NPC mitochondria in a time-dependent manner, whereas the inhibition of p53 mitochondrial translocation by pifithrin (PFT) significantly reduces compression-induced NPC apoptosis, suggesting that p53 may promote apoptosis by interacting with Bcl-2 family proteins through mitochondrial translocation [109]. At the same time, compressive loading promotes p53-regulated apoptosis-inducing protein 1 (p53AIP1) expression and induces p53-dependent apoptosis by interacting with Bcl-2 family proteins [270]. In addition, elevated p53 levels in NPC stimulate apoptosis by upregulating the expression of NDRG2 and regulating the development and DDR 1 (REDD1)/thioredoxin-interacting protein (TXNIP) complex, which in turn aggravates IDD [37, 271]. These studies confirm that the expression of p53 increases in degenerative IVD, leading to excessive apoptosis of IVD cells; this inhibition of p53 expression and activity may delay IDD by reducing apoptosis in IVD cells.

### p53 and cell senescence

As an age-related musculoskeletal disease, excessive cellular senescence in IVDs is an important feature of IDD. Cellular senescence can be divided into replicative and stress-induced senescence [272]. Replicative senescent cells accumulate during physiological or accelerated senescence processes and are aggravated by factors that promote the accumulation or inhibit the decomposition of senescent cells [273]. DNA damage, oxidative stress, and inflammation can significantly increase the burden of tissue cell senescence by inducing stress-related cell senescence [274–276]. In addition, senescent cells “infect” surrounding healthy cells through the paracrine effect of the senescence-associated secretory phenotype (SASP), which induces cellular senescence [277–279]. For instance, Moiseeva et al. [280] demonstrated that senescent cells can induce an inflammatory microenvironment that impedes the proliferation and regeneration of stem cells while alleviating the burden on senescent cells or neutralizing their inflammatory secretions to expedite regeneration in both juvenile and aged mice. High levels of SASP-associated MMPs have been shown to induce the shedding of autocrine ligands, making senescent cells less susceptible to immune surveillance and clearance [281]. These rapid accumulation, persistence, and amplification mechanisms lead to the accumulation of senescent cells in tissues, ultimately promoting the progression of IDD. p53-mediated cellular senescence is mainly derived from the DDR and can be induced by oxidative stress, inflammatory responses, telomere shortening, and oncogene activation [282, 283]. The DDR activates p53 through ATM and ATR, and the activated p53 subsequently undergoes phosphorylation and acetylation, which protects p53 from MDM2-mediated ubiquitin-dependent proteasome degradation [284]. After stabilization, p53 accumulates in the nucleus and activates the expression of effectors involved in the regulation of cellular senescence, among which p21 plays the most important role [285]. Once activated, p21 induces cell cycle arrest in the G1/S or G2/M phases by inhibiting apoptosis [286]. Histological analysis of human IVD specimens revealed that the proportion of senescence-associated β-galactosidase-positive cells was significantly higher in Pfirrmann grade IV/V than in grade I/II, and the expression of p53, p21, and Rb was high [66]. In addition, the expression of p53 mRNA and protein in rat degenerative IVD tissues was significantly increased [287]. In vitro, inflammation, oxidative stress, high glucose, and DNA damage can all induce senescence in IVD cells by activating the p53/p21/Rb signaling axis. In contrast, the downregulation of p53 expression can significantly improve cell senescence [68, 70, 101, 130, 183, 288, 289]. In addition, polo-like kinase 1 (PLK1), an evolutionarily conserved serine/threonine kinase, plays multiple roles in the cell cycle, including regulating cell proliferation and senescence [290]. Zhang et al. [67] showed significantly decreased expression of PLK1 in the NPCs of patients with IDD. Further functional experiments showed that inhibiting the activity of PLK1 in normal NPCs increased the activity of the p53/p21 signaling axis, inhibited cell proliferation, and induced cell senescence. In addition, N-acetylated proline-glycine-proline (N-Ac-PGP) is a collagen-derived tripeptide that plays an important role in inflammatory diseases by binding to CXC receptor 1/2 (CXCR1/2) [291]. N-Ac-PGP induces DNA damage and ROS accumulation in NPCs, which induces senescence by binding to CXCR1 and activating the p53/p21 signaling axis [128]. These results demonstrate that p53 is closely related to IVD cell senescence and that the inhibition of p53 expression and activation can effectively improve premature IVD cell senescence and delay IDD progression.

### p53 and ECM metabolism

As a central component of the stress response mechanism, p53 controls the safeguard mechanism against the accumulation of abnormal cells and their transformation by regulating DNA repair, cell cycle progression, cellular senescence, and death [292]. Recently, various cellular processes regulated by p53 have been shown to control glucose metabolism, oxidative stress, and ECM remodeling [293]. In a mouse model of dimethylnitrosamine (DMN) liver injury, the effect of DMN led to a dose-dependent increase in p53, accompanied by an increase in the expression of MMP9, 10, and 12 [294]. In addition, p53 overexpression significantly reduced the ECM of mouse fibroblasts [295]. Similarly, p53 overexpression significantly increased the expression of MMP13 and ADAMTS5 and inhibited the expression of SOX9 in mice with osteoarthritis, which significantly improved after the inhibition of p53 [296]. These studies suggest that p53 induces ECM remodeling by promoting the expression of matrix-degrading enzymes and inhibiting the expression of matrix proteins. In IDD, IL-1β significantly increased the expression level of p53 in NPCs, which resulted in the upregulation of mRNA and protein levels of MMP-3, MMP-13, ADAMTS-4, and ADAMTS-5 and the downregulation of Col II and Agg [103]. In addition, several studies have shown that abnormal activation of the NF-κB and JNK signaling pathways in IVD is an important factor contributing to IDD progression [143]. Activated NF-κB and JNK signaling pathways upregulate p53 expression, accelerating ECM degradation by promoting the expression of MMPs and ADAMTS and inhibiting the expression of Col II and Agg [71, 217]. Recently, Zheng et al. [103] reported that p53 binds to the promoter regions of prolyl endopeptidase to promote their transcription. Overexpression of prolyl endopeptidases impairs the ECM quality of NPCs by inhibiting the expression of Col II and Agg and promoting the expression of MMP3. p53 may have opposing roles in specific environments. Xiong et al. [72] reported that p53 plays an important role in maintaining NPC activity and integrity. Mechanistic studies have shown that high levels of p53 can promote ATP production by inhibiting the G6PD and pentose phosphate pathways. In addition, the expression of SOX9 and Col II in p53-normal controls was significantly higher than that in p53-knockdown NPCs. These results suggest that the selective activation of p53 downstream target genes under different stress induction conditions may lead to the differential expression of metabolism-related phenotypes within the IVD.

### p53 and oxidative stress

Oxidative stress can be located either upstream or downstream of p53. In normal IVD, NPCs exist in a hypoxic microenvironment, and high oxygen partial pressure enhances ROS production in NPC mitochondria [297]. At less than 20% oxygen partial pressure, the level of ROS in NPCs increases significantly, leading to p53 activation and cell senescence [298]. Furthermore, treatment of IVD cells with H_2_O_2_ resulted in a significant increase in intracellular ROS levels, which induced DDR and ATM activation, eventually leading to the activation and rapid accumulation of p53 [101, 299, 300]. These results suggest that increased ROS levels in IVD cells promote the expression and activation of p53. As a redox-sensitive transcription factor, p53 regulates various genes with antioxidant or pro-oxidative properties, affecting cellular redox homeostasis [301, 302]. The contradictory roles of p53 in oxidative stress regulation depend on various factors, including the type, intensity, and duration of stress [303]. Under physiological and mild stress conditions, p53 exerts its antioxidant activity by activating downstream antioxidant target genes, including TP53-inducible glycolysis and apoptosis regulator, sestrin 1/2 (SESN1/2), TP53-induced nuclear protein 1, and p21 [301]. These target genes reduce the overall level of intracellular oxidation by regulating cellular metabolism and maintaining mitochondrial function [304]. In addition, SESN1/2 and p21 interfere with Keap1–Nrf2 binding and enhance the stability and activity of Nrf2, thereby inhibiting intracellular oxidative stress [305, 306]. However, the phenomenon and related mechanism by which p53 inhibits oxidative stress in IDD remain unclear, and further research is needed. Under extensive and sustained stress, activated p53 exhibits pro-oxidative activity by turning on p53-inducible gene 3 and proline oxidase, leading to apoptosis by inducing the expression of Bax and PUMA [59, 304]. Additionally, TXNIP promotes intracellular oxidative stress by inhibiting thioredoxin reduction capacity [307]. In IDD, p53 expression increases with H_2_O_2_ treatment, and activated p53 further enhances oxidative stress [271]. These studies indicate a complex regulatory mechanism between p53 and oxidative stress. Further elucidation of the regulatory mechanisms between p53 and oxidative stress in IDD will be beneficial for developing potential therapeutic targets.

## p53-related targeted therapy

**Fig. 8:**
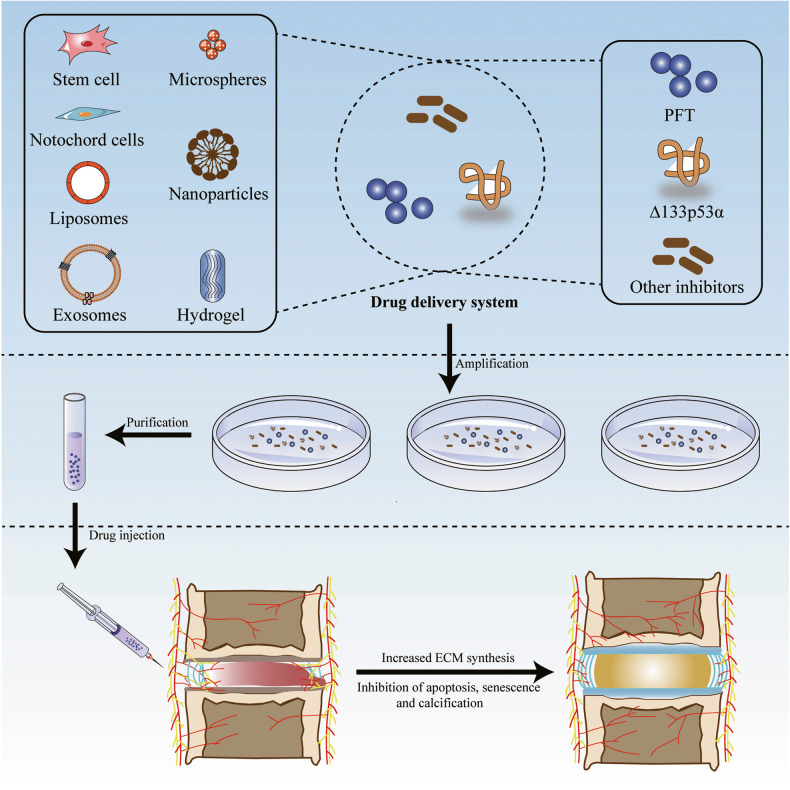
p53 inhibitors combined with drug delivery systems for the treatment of IDD. Top: Establishment of a drug delivery system for inhibitors of p53 function. Middle: Amplification and purification of the drug delivery system. Bottom: Injection of the drug delivery system.

The incidence and burden of IDD, an age-related disease, are increasing annually worldwide [3]. Currently, the treatment of IDD mainly includes conservative and surgical treatments, both aimed at relieving clinical symptoms. Therefore, the further exploration of the pathogenic mechanism of IDD and the adoption of targeted treatment strategies are current research hotspots. Recent studies have shown that p53 plays an important role in the occurrence and progression of IDD, and the inhibition of p53 overactivation in IVD may help delay the progression of IDD. However, extensive p53 inhibition may not be an applicable approach to alleviate IDD because the negative effects of this approach cannot be ignored. Therefore, p53-targeted functional inhibitors in combination with exosomes, biomaterials, and cellular therapies may yield greater benefits (Fig. 8).

### p53 inhibitor

In 1999, to reduce the serious side effects of p53-mediated chemotherapy and radiotherapy in cancer, Komarov et al. [308] isolated a small fraction of PFT to block the side effects caused by p53-dependent transcriptional activation. PFT protected mice from p53-related cell death induced by radiation and multiple cytotoxic drugs without promoting tumor formation [308]. Studies have confirmed that PFT can alleviate adriamycin-induced apoptosis in human umbilical vein endothelial cells by inhibiting basic and induced levels of the p53 protein [309]. In IDD, compression treatment induces NPC apoptosis by promoting the mitochondrial translocation of p53. In contrast, PFT significantly attenuates compression-induced NPC apoptosis and alleviates IDD progression by inhibiting p53 mitochondrial translocation [109]. These results suggest that PFT may delay the progression of IDD; however, further basic and clinical studies are needed to determine the optimal dose and route of administration.

### p53 homologous sequence

In addition to the typical full-length p53, TP53 produces at least 12 truncated subtypes through the alternative initiation of translation, the use of alternative promoters, and alternative splicing, which can positively or negatively regulate the activity and function of full-length p53 [281, 310]. Among these p53 isoforms, Δ133p53α and p53β are thought to be endogenous regulators of cellular senescence [311]. Under stress conditions, p53β forms a complex with full-length p53, which in turn enhances the transcriptional activities of the Bax and p21 promoters, suggesting that p53β acts synergistically with full-length p53 to induce apoptosis and senescence [281, 311]. ∆133p53α acts in contrast to p53β. ∆133p53α was abundantly expressed in early-passage normal human fibroblasts and was significantly reduced in late-passage and senescent cells, mainly due to excessive autophagic degradation of ∆133p53α in senescent cells [311]. In addition, in Hutchinson–Gilford progeria syndrome (HGPS) fibroblasts, ∆133p53α inhibited cellular senescence by downregulating the p53 senescence-associated genes p21 and miR-34a. ∆133p53α overexpression restored the replicative capacity of HGPS fibroblasts [312]. As mentioned above, the selective, dominant-negative effect of ∆133p53α on full-length p53 suggests that ∆133p53α may be a potent target for the inhibition of cellular senescence. However, the expression and role of 133p53α in IVD cells are unclear, and more studies are needed.

### Natural molecules

Numerous natural compounds have been investigated for their p53-inhibitory activities in the context of IDD. Naringin (Nar) and eupatilin (Eup), the primary flavonoids derived from *Citrus* and *Artemisia*, respectively, have been demonstrated to possess diverse biological effects [313, 314]. Both Nar and Eup regulate the expression of Col II, Agg, MMP-3, MMP-13, and ADAMTS-4 to maintain a high-quality ECM [68, 136]. Moreover, Eup suppresses TNF-α-induced senescence of NP cells through the downregulation of p21 and p53 expression [68]. Related mechanistic studies have shown that Nar and Eup can protect NPCs from damage by inhibiting the NF-κB/p53 signaling axis. In addition to Nar and Eup, quercetin (Que) and myricetin (Myr) belong to a family of natural flavonoids found in plants that exhibit anti-inflammatory, anti-aging, and antioxidant properties [315, 316]. Que and Myr can mitigate oxidative stress-induced IVD cell senescence by activating the SIRT1 signaling pathway, thereby inhibiting p53 expression [317, 318]. Morroniside (Mor), which belongs to the class of iridoid glycosides [319], has been reported to effectively attenuate H_2_O_2_-induced NP cell senescence by modulating the ROS-Hippo-p53 signaling pathway [101]. In vivo, Mor significantly ameliorated lumbar disc degeneration in rats after an 8-week treatment period. Furthermore, the inhibition of p53 by proanthocyanidins and resveratrol has been documented to effectively impede the progression of IDD [156, 320].

## Conclusions and prospects

IDD is the initial step in the evolution and progression of a range of degenerative spinal disorders and is a major cause of LBP and disability, the prevalence of which increases with age [321]. Currently, the main treatment for IDD is to control the clinical symptoms through physiotherapy, oral drugs, and surgery; however, it is not to prevent the progression of IDD or reverse its degeneration by treating the causes of degenerative disease [322]. However, these methods lead to a high recurrence rate, treatment cost, and risk of adjacent IVD degeneration [323]. Therefore, the pathogenic mechanisms of IDD and the development of targeted therapeutic approaches are major clinical issues that must be addressed. Transcription factor p53 plays an important role in malignant tumors, cardiovascular diseases, neurodegenerative diseases, and osteoarticular diseases by regulating cell cycle progression, senescence, apoptosis, angiogenesis, DNA repair, and cell metabolism [44, 45, 138, 324]. Recent studies have shown that p53 activation and signal transduction maintain homeostasis in IVD. p53 reduces the number of normal IVD cells by activating intrinsic death mechanisms and senescence-related genes in IVD cells. In contrast, p53 alters the microenvironment where IVD cells survive by promoting the expression of a metabolic phenotype characterized by SASP, leading to an imbalance between anabolic and catabolic cellular metabolism and a decrease in IVD ECM content. The combined action of these two factors promotes abnormal changes in the organizational structure of the IVD, eventually leading to IDD. Current research suggests that inhibiting the activation of p53 and its downstream signaling pathways delays the progression of IDD. However, a few studies have shown that p53 plays an important role in maintaining the activity and functional integrity of NPCs under low-glucose conditions [72]. This suggests that p53 may play different roles at different stages of IDD. Further studies are required to elucidate the role of p53.

Studies have shown that p53 responds differently based on the severity and duration of stimulation. Mild and transient stimuli induce the repair of cell damage and transient growth arrest. In contrast, severe and sustained stimuli can lead to cell senescence and apoptosis [325]. Currently, studies on p53 in IDD mainly focus on the dynamic changes and role of p53 in late degeneration and severe stress. However, the dynamic changes and activation of related signaling pathways of p53 in early-stage IVD and sublethal stress have not yet been elucidated. Therefore, future studies should focus on the expression pattern of p53 in the early stages of IDD and its role in related phenotypic changes to explore targeted treatment strategies.

In addition, studies on the role of p53 in IDD are still in the exploratory stage, with most studies focusing on rodent models. However, there are still certain species-specific differences in the shape, size, and biochemical composition of the IVDs and the anatomical structure of the spine between rodents and large mammals [326], which makes many current studies inapplicable to human IDD, making it difficult to guide treatment. Therefore, selecting an appropriate animal model is significant for future basic research and clinical applications. Therefore, sheep may be more suitable than rodents for studying human IDD. Sheep IVDs have a shape and size similar to human IVDs and do not exhibit the persistence of notochord cells with age [323]. Additionally, current studies have focused on the NP, and future treatments should focus on the synergistic recovery of the NP, AF, and CEP. Simultaneously, because the progression of IDD is a chronic process, the long-term efficacy of related treatment strategies must be evaluated.

In conclusion, the molecular mechanism underlying the potential role of p53 in IDD has not yet been fully elucidated and requires further investigation. Future research should focus on the role of p53 in early IDD, which appears to be crucial for further elucidation of its pathogenesis and the development of targeted therapies.
